# Circular Letters

**Published:** 1914-06

**Authors:** 


**Circular Letters.** Please remember that the circular letter is a nuisance to the busy man and that it is the worst competitor with legitimate advertising in periodicals. Every answer to a circular tends to deteriorate and advance the cost of periodical literature of every kind. We do not, of course, allude to sending through the mails or by express, bulky catalogues, etc., obviously too extensive for ordinary advertising space.

